# Circulating galectin-3 promotes tumor-endothelium-adhesion by upregulating ICAM-1 in endothelium-derived extracellular vesicles

**DOI:** 10.3389/fphar.2022.979474

**Published:** 2022-10-25

**Authors:** Lei Wang, Dan-Dan Du, Zong-Xue Zheng, Peng-Fei Shang, Xiao-Xia Yang, Chao Sun, Xiao-Yan Wang, Ya-Jie Tang, Xiu-Li Guo

**Affiliations:** ^1^ Department of Pharmacology, Key Laboratory of Chemical Biology (Ministry of Education), School of Pharmaceutical Sciences, Shandong University, Jinan, China; ^2^ State Key Laboratory of Microbial Technology, Shandong University, Qingdao, China

**Keywords:** galectin-3, vascular endothelial cell, extracellular vesicles, ICAM-1, triple negative breast cancer

## Abstract

The adhesion of tumor cells to vascular endothelial cells is an important process of tumor metastasis. Studies have shown that tumor could educate vascular endothelial cells to promote tumor metastasis through many ways. However, the effect of tumor cells on the functions of vascular endothelial cells-derived extracellular vesicles (H-EVs) and the mechanisms underlying their effects in tumor-endothelium adhesion in metastasis remain mysterious. In this study, we found that H-EVs promoted the adhesion of triple negative breast cancer cell to endothelial cells and cirGal-3 enhanced the adhesion-promoting effects of H-EVs. The underlying mechanism was related to the upregulation of glycolysis in endothelial cells induced by cirGal-3 which led to the increase of the ICAM-1 expression and its transmission to MDA-MB-231 cells by H-EVs. Targeting of cirGal-3 or glycolysis of vascular endothelium in breast cancer therefore represents a promising therapeutic strategy to reduce metastasis.

## Graphical Abstract

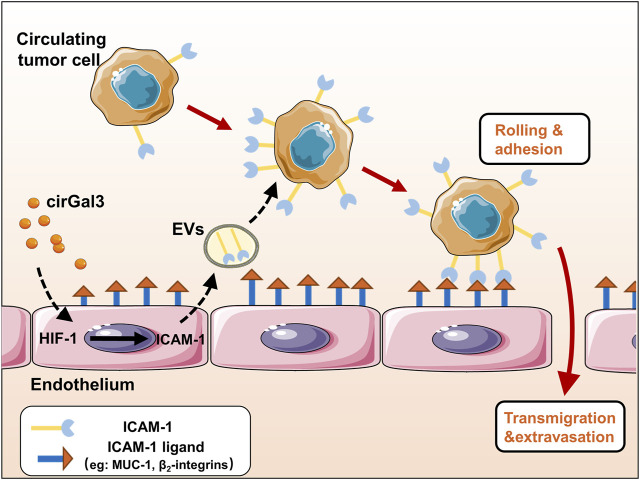

## Introduction

Endothelial cell-derived extracellular vesicles (EVs) have been shown to be involved in the development of atherosclerosis, cardiomyopathy, ischemia-reperfusion injury and tumor in recent years (Paone et al., 2018; Mccann et al., 2019; Berezin and Berezin, 2020). Vascular endothelial cells are critical components in the process of tumor metastasis (Reymond and Ridley, 2013; Mashreghi et al., 2021). Cancer cells attach and cross the endothelial cells that line blood vessel to form metastasis. During this process, cancer cells often secrete cytokines to increase endothelial barrier permeability, resulting in the promotion on the efficiency of cancer cell extravasation. At the same time, the educated vascular endothelial cells also secrete a variety of metastasis-promoting cytokines mediated by EVs (Yu, 2010; Maishi and Hida, 2017). Studies showed that endothelium-derived EVs could regulate the proliferation of cancer cells by delivering miR-503 (Bovy et al., 2015) or miR-126 (Monaco et al., 2019). However, the regulatory mechanism of endothelium-derived EVs and their effects on tumor metastasis still remains unknown.

Galectin-3 (Gal-3) is a widely expressed multi-functional protein, which plays different roles by binding with va rious intracellular, membrane and extracellular ligands (Jin et al., 2021). Also, it has been shown to play a role in many aspects of tumor development, such as proliferation, anti-apoptosis, metastasis, and angiogenesis (Farhad et al., 2018). Compared with that in localized tumors, circulating Gal-3 (cirGal-3) in patients with metastatic tumor were significantly elevated. These evidences indicated that cirGal-3 might play a crucial role in tumor metastasis (Iurisci et al., 2000). Recently, we have shown that tumor hypoxia microenvironment induced the secretion of Gal-3 from tumor associated macrophage, leading to the increase of Gal-3 level in serum (Wang et al., 2020). Also, we found that the increased Gal-3 promoted the adhesion of tumor cells to vascular endothelial cells by regulating E/N-cadherin and CD44 expression in tumor cells (Cao et al., 2018). However, whether cirGal-3 could regulate the secretion of metastasis-promoting cytokines mediated by EVs from endothelial cells still remains unknown.

Triple negative breast cancer (TNBC) is the breast cancer with negative expression of estrogen receptor alpha, progesterone receptor and epidermal growth factor receptor. It accounts for 10–20% of breast cancer incidence, yet is responsible for 30% of all breast cancer death. TNBC is characterized by high rate of proliferation and metastasis, and there is no special target. In this study, we reveal that the increased cirGal-3 induced the secretion of intercellular cell adhesion molecule-1 (ICAM-1) in EVs from human umbilical vein endothelial cells (HUVECs), leading to enhance the adhesion of TNBC MDA-MB-231 cells to vascular endothelial cells and promote tumor metastasis.

## Material and methods

### Reagents

Gal-3 (Abcam, MA, United States); 2-Deoxy-D-glucose (Meilun Biological, Dalian, China).

### Cell lines

Human triple negative breast cancer cell line MDA-MB-231 was obtained from cell bank of the China Science Academy (Shanghai, China). Human umbilical vein endothelial cells (HUVECs) were purchased from ATCC (Rockvile, United States). The MDA-MB-231cell line labeled with luciferase (MDA-MB-231-luc) was supplied by Caliper (Hopkinton, United States). Cells were cultured in RPMI-1640 medium (Gibco, CA, United States) supplemented with 10% fetal bovine serum (Gibco, CA, United States) at 5% CO_2_, 37°C.

### Western blot analysis

Western blot was performed as previously described (Wang et al., 2020). Shortly, proteins were separated by SDS-PAGE and were electrically transferred to PVDF membrane (Millipore, Billerica, United States). The membranes were blocked for 4 hour with 5% (w/v) skim milk, then incubated with primary antibodies against human CD9 (#AF5139, affinity, United States), CD63 (#AF5117, affinity, United States), ICAM-1 (10831-1-AP, Proteintech, China) and anti-β-actin (#ZF-0313, ZS Bio, China) overnight, and incubated with second antibodies goat anti-mouse or anti-rabbit IgG. Membranes were visualized with enhanced chemiluminescence detection.

### Extraction of extracellular vesicles of human umbilical vein endothelial cells

In brief, when the cell density reaches about 70%–80%, the culture medium was changed to RPMI-1640 medium containing 10% extracellular vesicles-free FBS (Vivacell, Sartorius, Germany) at 37°C, 5% CO_2_. After 48 h, the cell culture medium was harvested to extract extracellular vesicles in strict accordance with manufacturer’s instructions of Exoquick-TC exosome isolation reagent (SBI, CA, United States). The precipitate was finally resuspended with different solutions, according to the downstream experiment. In detail, PBS filtered with a 0.22 μm filter was added to resuspend the precipitate for observing the morphology of extracellular vesicles by transmission electron microscope; RIPA lysate was added for Western blot assay; Trizol was added for qRT-PCR assay.

### Transmission electron microscopy

First, 10 µl of extracellular vesicles was added dropwise to the copper mesh surface of the carbon support membrane, and excess solvent was blotted away with filter paper. Imaging of extracellular vesicles was detected by projection electron microscope (Hitachi HT-7700, Tokyo, Japan) at 100 kv after drying at room temperature.

### Extracellular vesicles labeling and tracking

The collected cell culture medium was centrifuged at 300 × g at 4°C for 10 min. After filtering through a 0.22 µm filter, the supernatant was added with 1 µl Dio green fluorescent dye to label extracellular vesicles in medium at 37°C. After that, extracellular vesicles were extracted following strictly the manufacturer’s instructions of Exoquick-TC exosome isolation reagent (SBI, CA, United States). The labeled extracellular vesicles were incubated with MDA-MB-231 cells for 6 h. Then cells were washed three times with PBS and fixed with methanol glacial acetic acid (3:1) for 20 min. Finally, cells were stained with DAPI for 30 min, and photographed with Nikon TE 2000-U microscope (NIKON, Tokyo, Japan).

### Cell adhesion

MDA-MB-231 were incubated with extracellular vesicles for 24 h and stained with Dio green fluorescent dye for 20 min at 37°C. After washing with PBS, MDA-MB-231 were released and applicated to the HUVECs monolayer cultured in 24-well plates for 30 min at 37°C. The non-adherent tumor cells were removed from the 24-well plates by gently washing with PBS, and the fluorescently labeled cells remaining on the endothelial monolayer were observed under fluorescence microscope.

### Glucose uptake assay

Glucose concentration was detected by Glucose Assay Kit (NJJCBIO, Wuhan, China). The glucose uptake is calculated by subtracting the amount of glucose remaining in the cell culture medium from the amount of glucose in the fresh medium.

### Quantitative real-time PCR

qRT-PCR was performed as previously described (Wang et al., 2020). In brief, RNA was extracted from HUVECs by using TRIzol reagent (Invitrogen, California, United States) and reverse transcribed with RT-PCR Kit (Toyobo, Osaka, Japan) according to the manufacturer’s instructions. Then reverse transcribed with RT-PCR Kit (Toyobo, Osaka, Japan). Target gene sequences were purchased from Jinan Boshang Biotechnology Co., Ltd, the primers sequences as below: ICAM-1 (5′-3′): GCC​TGG​GAA​CAA​CCG​GAA​GGT​G.

### Correlation analysis

The TCGA RNA-seq data for breast invasive carcinoma (BRCA) were downloaded from the TCGA website. For the correlation analyses between gene expression, Spearman correlation coefficients were calculated.

### Tumor metastasis mouse model and IVIS imaging

1×10^6^/100 μl/each mouse H-EVs‐educated MDA-MB-231-luc cells with different treatments were intravenously injected to each balb/c nude mice (female, 5-week-old) by tail vein (Control group: cell was treated with PBS for 24 h; H-EVs group: cell was treated with H-EVs for 24 h; HGal3-EVs group: cell was treated with EVs from HUVECs treated with Gal3 for 24 h; HGal3-2DG-EVs group: cell was treated with EVs from HUVECs treated with Gal3 and 2DG for 24 h). The body weight of mice was recorded every 3 days. Mice were imaged using an *in vivo* imaging system (Caliper Life Sciences, MA, United States). At the end of experiments, the lungs of mice were removed and photographed with Zeiss STEMI 508 stereomicroscope (Zeiss, Wuertenburg, Germany).

### Statistical analysis

The result are expressed as mean ± SD. A *p*-value < 0.05 was considered statistically significant. Statistical analysis was performed using the GraphPad Prism6.

## Result

### Gal-3 treated human umbilical vein endothelial cells derived H-EVs entered MDA-MB-231 cells to promote cell adhesion to human umbilical vein endothelial cells and migration

**FIGURE 1:**
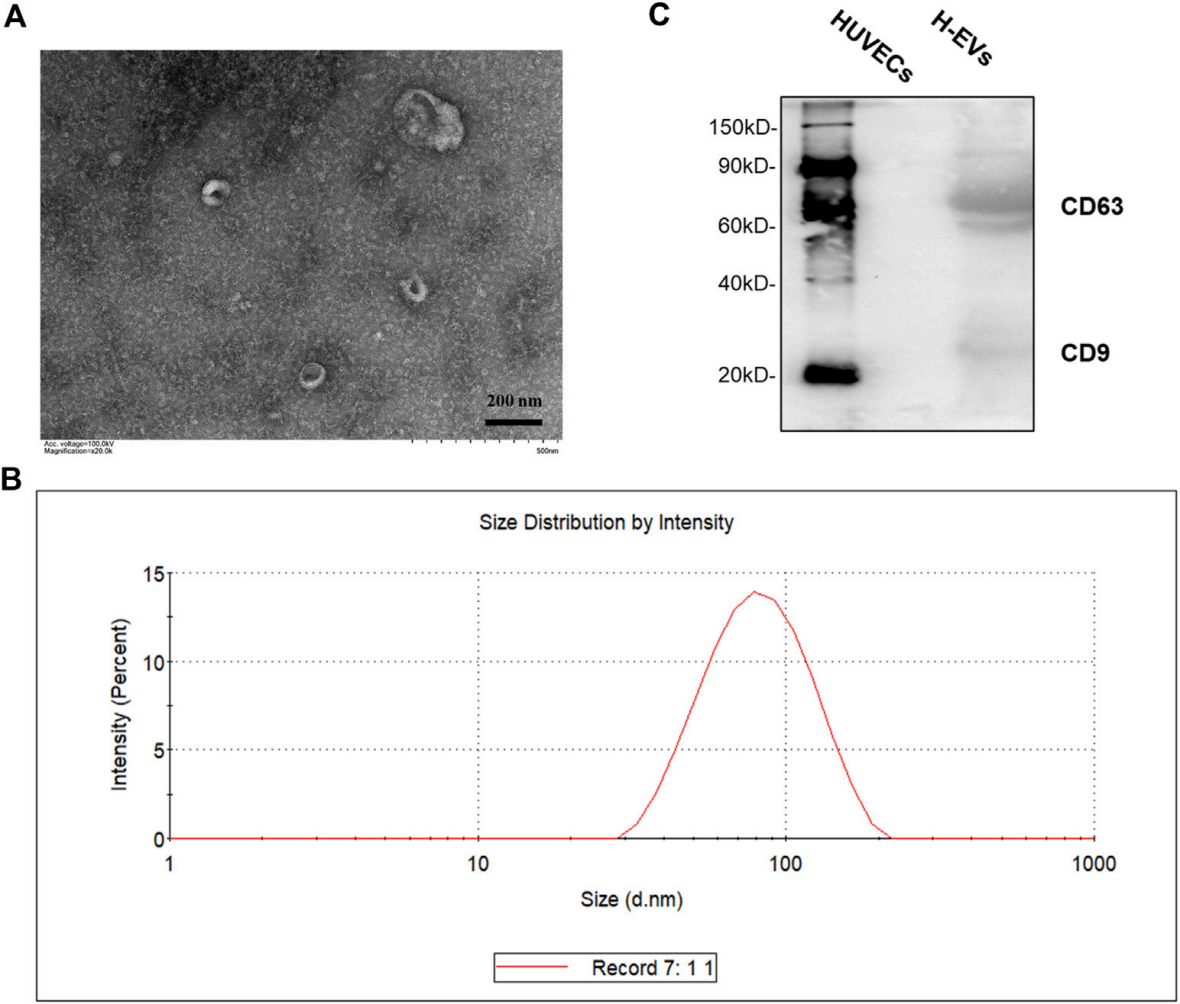
Characterization of HUVECs derived EVs (H-EVs). **(A)** Examination of isolated EVs by TEM. **(B)** The size distribution of exosomes was determined by DLS. **(C)** The expressions of EVs biomarkers CD9, CD63 in EVs samples were detected by Western blot.

EVs were isolated from the culture medium (CM) from HUVECs. As shown in Figures 1A,B, transmission electron microscope (TEM) visualized typical rounded particles with a diameter of <200 nm, and dynamic light scattering (DLS) showed that the EV size distribution was from 50 to 150 nm. In addition, EVs markers CD9 and CD63 was significantly expressed in EVs, while there was no expression in HUVECs which was detected by Western blotting (Figure 1C).

**FIGURE 2:**
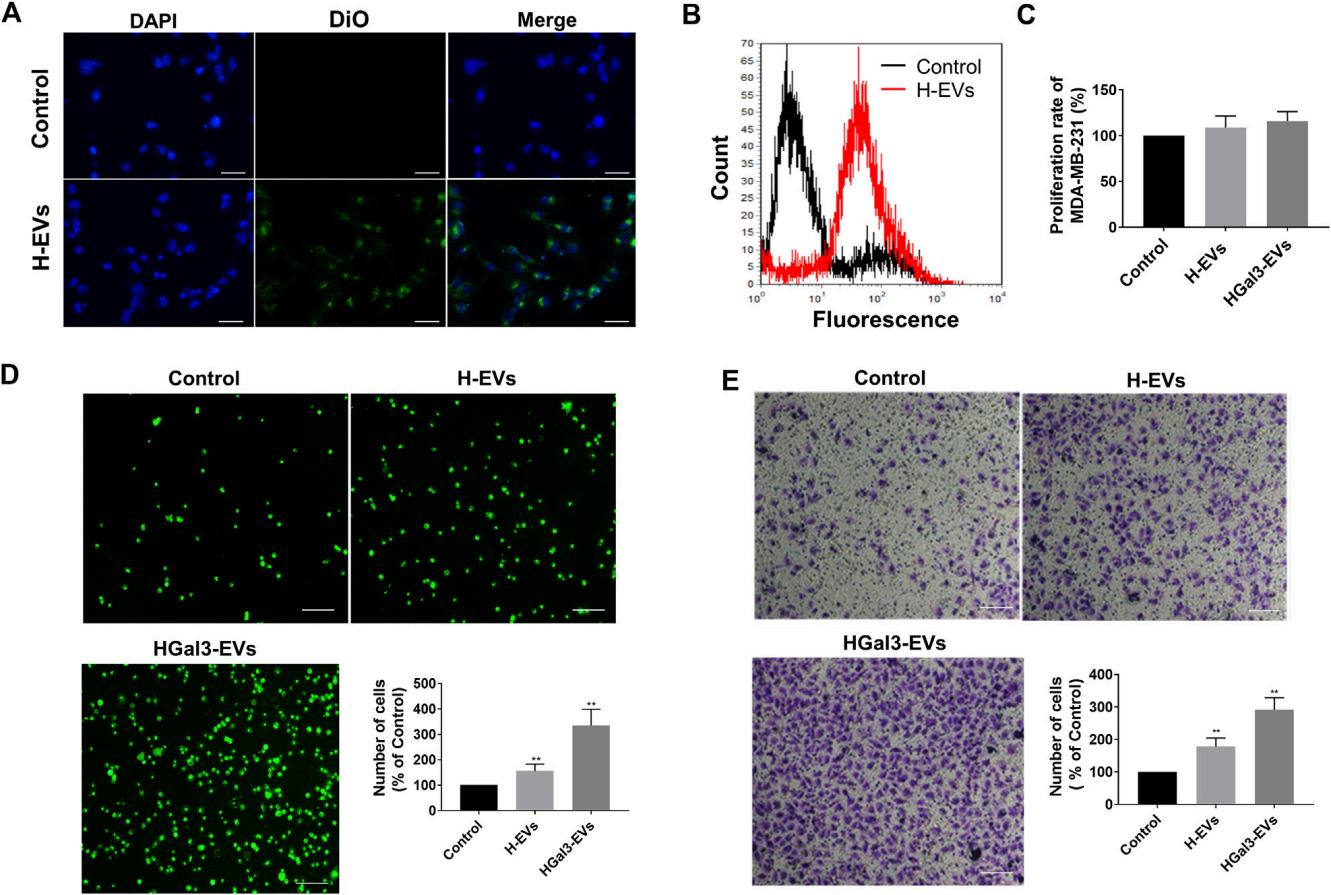
Gal-3 treated HUVECs derived H-EVs entered MDA-MB-231 cells to promote migration and cell adhesion to HUVECs. **(A)** Fluorescent image of the internalization of Dio-labeled H-EVs in MDA-MB-231 cells (magnification, ×200, bar = 200 μm). **(B)** The internalization of Dio-labeled was detected by flow cytometry experiment. **(C)** The effect of H-EVs on the proliferation of MDA-MB-231 cells was detected by CCK-8. Control: PBS; H-EVs: EVs from HUVECs; HGal3-EVs: EVs from HUVECs treated with 1 ng/ml Gal-3 for 24 h. **(D)** The effect of H-EVs on the adhesion of MDA-MB-231 cells to HUVECs was detected by adhesion assay (magnification, ×100,bar = 100 μm). **(E)** The effect of H-EVs on the migration of MDA-MB-231 cells was detected by Transwell assay (magnification, ×100, bar = 100 μm). Data are presented as the means ± S.E.M. **p < 0.01, *v.s*. control group.

To determine the effects of H-EVs on the progression of MDA-MB-231 cells, we firstly investigated whether H-EVs could be transferred to MDA-MB-231 cells. As shown in Figures 2A,B, the H-EVs stained with DiO dye could rapidly enter MDA-MB-231 cells. Then we investigated the effects of H-EVs on the proliferation, adhesion and migration of MDA-MB-231 cells. Results showed that H-EVs had no significant effect on the proliferation of MDA-MB-231 cells (Figure 2C), while obviously increased the endothelial adhesion and migration of MDA-MB-231 cells. Moreover, EVs isolated from HUVECs treated with exogenous Gal-3 for 24 h (HGal3-EVs) enhanced dramatically the pro-tumor adhesion and migration function of H-EVs (Figures 2D,E). These findings indicated that cirGal-3 could act on HUVECs to promote the endothelial adhesion and migration of breast cancer cells via H-EVs.

### Gal-3 increased the glycolysis of human umbilical vein endothelial cells to promote the effect of H-EVs on cell adhesion

**FIGURE 3:**
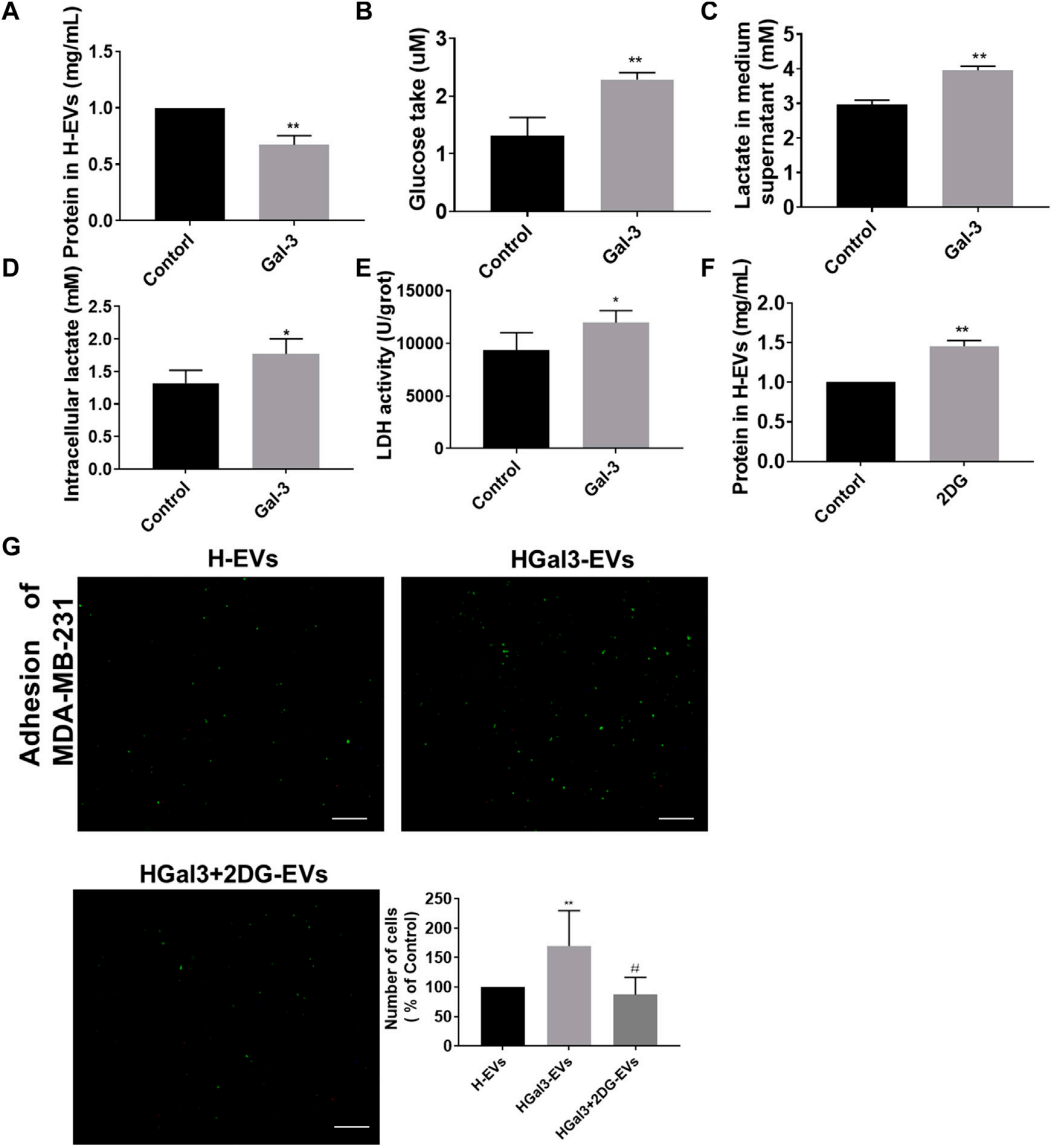
Gal-3 increased the glycolysis of HUVECs to promote the effect of H-EVs on cell adhesion. **(A)** Effect of 1 ng/ml Gal-3 on the protein concentration in H-EVs for 24 h was detected by BCA protein kit. **(B)** Effect of Gal-3 on the glucose uptake of HUVECs was detected by glucose detection kit. **(C,D)** Effect of Gal-3 on the intracellular lactate content and lactate secretion of HUVECs were detected by lactate detection kit. **(E)** Effect of Gal-3 on LDH activity in HUVECs was detected by LDH kit. **(F)** Effect of 4 nM 2-DG on the protein concentration in H-EVs for 24 h was detected by BCA protein kit. **(G)** The effect of HGal3-EVs on the adhesion of MDA-MB-231 cells to HUVECs was detected by adhesion assay (magnification, ×100, bar = 100 μm). Data are presented as the means ± S.E.M. *p < 0.05, **p < 0.01, *v.s*. control group; ^#^ *p* < 0.01, *v.s*. HGal3-EVs group.

EVs transported amounts of proteins, lipids, and nucleic acids to other cells. We unexpectedly noticed that Gal-3 reduced total protein amount in H-EVs (Figure 3A). As Gal-3 has been reported to be involved in glucose metabolism, we wonder whether Gal-3 regulates protein expression by regulating glucose metabolism. Results showed that the glucose uptake, intracellular lactate content, lactate secretion and intracellular LDH levels of HUVECs were significantly increased after treatment with exogenous Gal-3 (Figures 3B–E), suggesting that Gal-3 induced the glycolysis of HUVECs. After that, we used the glycolysis inhibitor 2DG to confirm that role of glycolysis in the decrease of total proteins amount in H-EVs and tumor-endothelial adhesion induced by Gal-3. The results showed that 2DG could promote the expression of total protein amount in H-EVs (Figure 3F). Moreover, 2DG reduced the promoting effect of Gal-3 on tumor-endothelial adhesion induced by H-EVs (Figure 3G), confirming that Gal-3 increased the glycolysis of HUVECs to promoted tumor-endothelial adhesion by H-EVs.

### Gal-3 upregulates the expression of intercellular cell adhesion molecule-1 in H-EVs to promote tumor-endothelial adhesion

**FIGURE 4:**
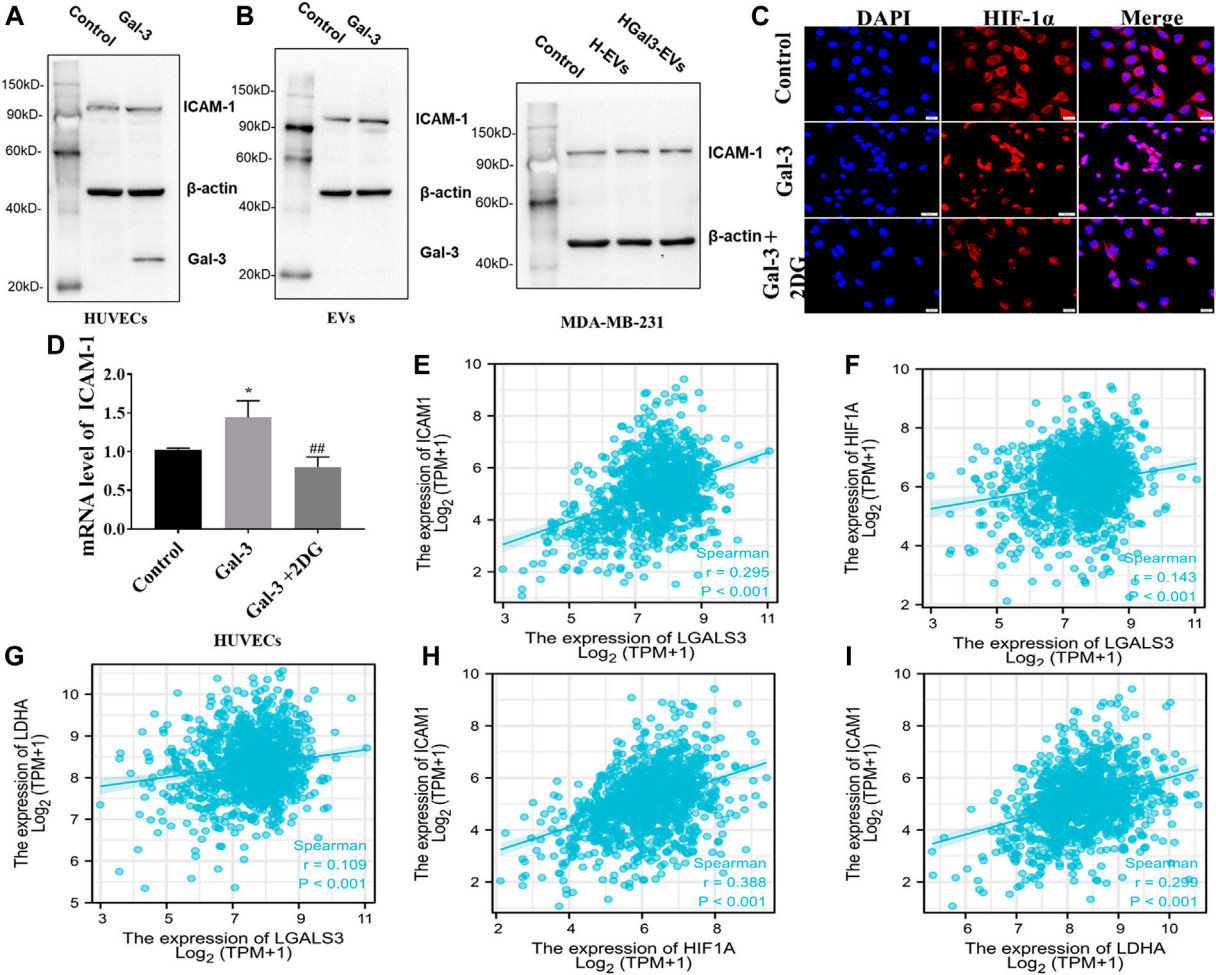
Gal-3 upregulates the expression of ICAM-1 in H-EVs to promote tumor-endothelial adhesion. **(A)** Expression of ICAM-1 in H-EVs and HUVECs after incubated with 1 ng/ml Gal-3 for 24 h was detected by Western blot. **(B)** Effect of H-EVs and HGal3-EVs on the expression of ICAM-1 in MDA-MB-231 cells was detected by Western blot. **(C)** Effect of 2-DG on Gal-3-induced HIF-1α nucleation in HUVECs were assessed by immunofluorescence (magnification, ×100, bar = 200 μm). **(D)** Effect of 4 nM 2-DG on the increased mRNA expression of ICAM-1 in HUVECs induced by 1 ng/ml Gal-3 was detected by RT-PCR. **(E–I)** Lgals3, ICAM1, HIF-1A and LDHA expression from TCGA RNA-seq data were examined for correlation analyses. Correlations between them in BRCA (*n* = 1222) were calculated by Spearman rank correlation test. Data are presented as the means ± S.E.M. *p < 0.05, *v.s.* control group; ^##^ *p* < 0.01, *v.s.* HGal3-EVs.

ICAM-1, an important member of the immunoglobulin superfamily and an adhesion receptor, has been reported to be expressed on the tumor cells and HUVECs to participate tumor-endothelial adhesion (Bui et al., 2020). Whether the promoting effect of Gal-3 on tumor-endothelial adhesion induced by H-EVs was related to ICAM-1 was investigated. Result showed that exogenous Gal-3 entered HUVECs to promote the expression of ICAM-1 in HUVECs which was loaded into H-EVs, while Gal-3 could not enter H-EVs (Figure 4A). Compared with H-EVs, HGal3-EVs increased the expression of ICAM-1 in MDA-MB-231 cells (Figure 4B), suggesting that Gal-3 promoted tumor-endothelial adhesion by increasing the expression of ICAM-1 in H-EVs which could enter tumor cells. In addition, 2DG inhibited the Gal-3-induced nucleation of ICAM-1 transcription factor HIF-1α and ICAM-1 mRNA expression in HUVECs (Figures 4C,D), suggesting that the increase of glycolysis level induced by Gal-3 led to the increase of ICAM-1 expression.

To identify potential correlations between Gal-3, glycolysis and ICAM-1, TCGA datasets was used. Spearman correlation analysis demonstrated positive correlation in LGALS3 (Gal-3 gene), ICAM1, HIF-1A and LDHA (glycolysis related gene) (Figures 4E–I). Therefore, these observations support the conclusion that the increase of glycolysis level induced by Gal-3 led to the increase of ICAM-1 expression.

### HGal3-EVs promoted lung metastasis of MDA-MB-231 cells by enhancing the glycolysis of human umbilical vein endothelial cells in nude mice

**FIGURE 5:**
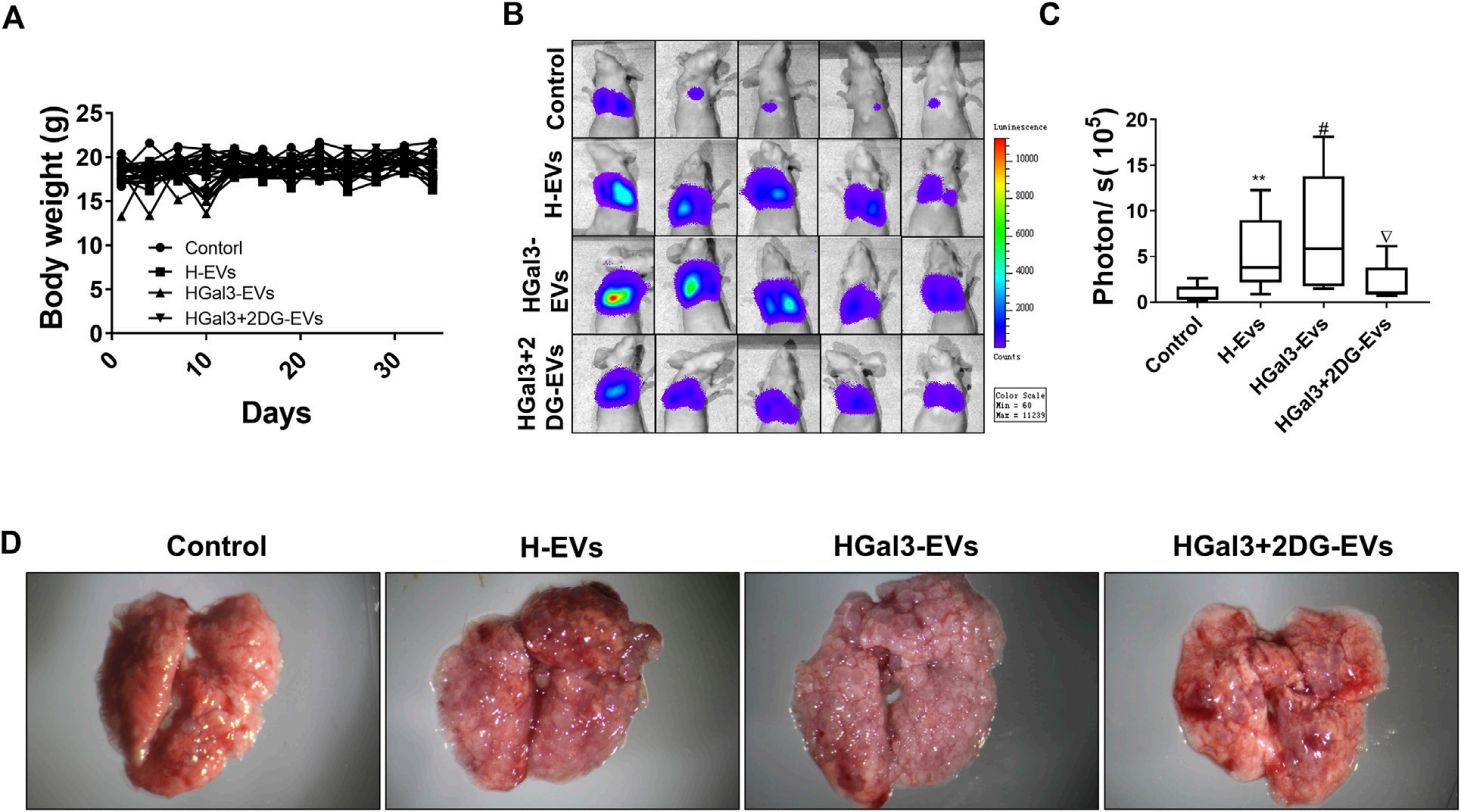
HGal3-EVs promoted lung metastasis of MDA-MB-231 cells by enhancing the glycolysis of HUVECs in nude mice. **(A)** The body weight of mice was recorded every 3 days for 5 weeks. **(B)** The lung metastasis of MDA-MB-231 cells was observed using an *in vivo* imaging system. **(C)** Quantitative bioluminescence intensity of pulmonary metastatic cells. **(D)** Representative lung tissue in each group. All data are presented as means ± SD, *n* = 5, **p < 0.01 *v.s.* control group; ^#^ *p* < 0.05, *v.s.* H-EVs group; ^∇^ *p* < 0.05, *v.s*. HGal3+2DG-EVs.

To investigate the enhancement of Gal-3 on the pro-tumor metastasis function of H-EVs *in vivo*, MDA-MB-231-luc cells treated with H-EVs, or HGal3-EVs, or HGal3+2DG-EVs were injected into nude mice via tail vein. After 5 weeks, we found that there was no significant difference in body weight among four groups (Figure 5A). Compare with control group, MDA-MB-231 cells treated with either H-EVs or HGal3-EVs generated more bioluminescence intensity and the number of pulmonary nodules (Figures 5B–D). Moreover, HGal3-EVs treatment showed stronger intensity (Figure 5C) and much more nodules (Figure 5D) than H-EVs treatment. Furthermore, HGal3+2DG-EVs treatment reduced significantly the bioluminescence intensity and the number of pulmonary nodules of MDA-MB-231 cells in comparison with HGal3-EVs treatment (Figures 5B–D). These findings indicated that Gal-3 induced the tumor metastasis of breast cancer cells via H-EVs, and the underlying mechanism was related closely to its regulation on the glycolysis of HUVECs.

## Discussion

As EVs has been reported to involve in tumor progression (Goričar et al., 2021; Wu et al., 2021; Thakur et al., 2022), the detection of EVs as diagnostic and diagnostic biomarkers of tumor (Thakur et al., 2020a; Qu et al., 2021; Xu et al., 2021)and EVs based drug delivery systems (Thakur et al., 2020b; Gaurav et al., 2021) has attracted extensive attention. Further research on its regulatory effects on tumor metastasis and underlying mechanism will be beneficial to its clinical application. Saussez et al. reported that cirGal-3 in patients with metastatic tumor were obviously higher (Saussez et al., 2008). In the present study, we investigated the effect of Gal-3 on the pro-tumor function of EVs from vascular endothelial cells and elucidated the underlying mechanism. We first found that H-EVs induced the adhesion of breast cancer cells to HUVECs, and Gal-3 enhanced the adhesion-promoting effect of H-EVs.

EVs are cell-derived small particles with a stable bilayer membrane structure (Loch-Neckel et al., 2022). The diameter of EVs were approximately 50–150 nm with a “cup” shape morphology (Liu et al., 2020). Currently, several techniques are being used for the detection and characterization of EVs, including nanoparticle tracking analysis, electron microscopy, immunogold-electron microscopy, atomic force microscopy, localized surface plasmon resonance biosensor, etc (Thakur et al., 2017; Thakur et al., 2017; Qiu et al., 2018; Thakur et al., 2020c; Thakur et al., 2021). Our observation is consistent with these reports. Additionally, we detected the expression of H-EV surface markers CD9, CD63 to confirm that the extracted vesicles from HUVECs are EVs.

Galectin-3 is a multifunctional protein, which plays different roles in different stages of different tumors. Circulating galectin-3 in patients with metastatic tumor were significantly elevated. In the study, we noticed that galectin-3 could reduce the secretion of EVs which is quantified by the total protein expression in H-EVs and the expression of ICAM-1 in EVs. The protein expression in EVs is closely related to tumor development (Qu et al., 2021). ICAM-1 is an adhesion protein expressed on the surface of endothelial cells, monocytes, lymphocytes and tumor cells (Ramos et al., 2014). Higher serum ICAM-1 concentration has been shown in various cancers and mediated hematogenous metastasis, lymphatic metastasis and immune escape of tumor cells (Alexiou et al., 2001). ICAM-1 can bind to a variety of cell surface proteins, such as integrin β2, mucin 1 (MUC1), thus participating in intercellular binding (Bernier et al., 2011; Sökeland and Schumacher, 2019). Tumor cell can also use leukocytes as linker cells to adhere to the endothelium by ICAM-1. It has been shown that breast tumor express ICAM-1 and interacts with integrin β2 on neutrophil granulocytes. The neutrophil granulocytes then, *via* integrin β2, bind to the ICAM-1 on endothelial cells (Strell et al., 2007). In this study, we found that HUVECs delivered ICAM-1 to MDA-MB-231 cells through EVs, then increased the adhesion of MDA-MB-231 cells to HUVECs. Gal-3 upregulated the expression of ICAM-1 in HUVECs and H-EVs to promote the pro-adhesion of MDA-MB-231 to HUVECs mediated by H-EVs. These findings indicate that Gal-3 induces ICAM-1 expression in the HUVECs and deliver it to breast cancer cells through EVs, resulting in the promotion of tumor-endothelial adhesion.

It has been reported that glycolysis could reduce the secretion of EVs from tumor cell lines UMSCC47, PCI-13 or Mel526 (Ludwig et al., 2020). Moreover, inhibition of glycolysis reduced the expression of ICAM-1 in HUVECs to the adhesion of tumor and endothelial cells (Qi et al., 2019). Gal-3 was found to participate in the glycolysis by regulating the expression of GLUT-1 (Li et al., 2020; Wang et al., 2021), suggesting that Gal-3 might reduce the secretion of H-EVs and induce ICAM-1 expression of H-EVs by promoting glycolysis of HUVECs. Hence, we introduced glycolysis inhibitor 2DG and confirmed that glycolysis induced by Gal-3 in HUVECs was involved in the decrease of secretion of H-EVs and the increase of ICAM expression in H-EVs. Moreover, the pro-adhesion effect of Gal-3 was suppressed by 2DG, suggesting that glycolysis induced by Gal-3 in HUVECs was involved in the pro-adhesion effect. Furthermore, TCGA dataset from 1222 BRCA patients showed the positive correlation between Gal-3 gene expression, ICAM1 expression and glycolysis related gene expression using Spearman correlation analysis.

We further investigated the effect of H-EVs on the tumor metastasis in mice tumor models. The results showed that H-EVs promoted lung metastasis of breast cancer cells, and Gal-3 enhanced this effect. Moreover, the pro-metastasis effect of HGal3-EVs was suppressed by 2DG, suggesting that targeting glycolysis of vascular endothelium is a potential anti-tumor metastasis strategy.

The present study has several limitations. In this study, we focused on the effect of H-EVs on the adhesion of circulating tumor cells in tumor metastasis. However, as tumor metastasis is a complex process, H-EVs may affect multiple steps of tumor metastasis by multiple ways. In future study, we will screen the miRNA and LncRNA in the H-EVs that induced tumor metastasis by RNA-Seq.

In conclusion, we found that H-EVs promoted the adhesion of MDA-MB-231 cells to HUVECs, which was enhanced by cirGal-3. The underlying mechanism was closely related to the upregulation of ICAM-1 expression in HUVECs by Gal-3 and the delivery of ICAM to MDA-MB-231 cells through H-EVs, resulting in the increased metastasis of breast cancer. Targeting of cirGal-3 or glycolysis of vascular endothelium in therefore represents a promising therapeutic strategy to reduce metastasis of triple negative breast cancer.

## Data Availability

The datasets presented in this study can be found in online repositories. The names of the repository/repositories and accession number(s) can be found in the article/Supplementary Material.
